# Application of nanoparticles in the diagnosis and treatment of chronic kidney disease

**DOI:** 10.3389/fmed.2023.1132355

**Published:** 2023-04-17

**Authors:** Kaibi Yang, Yiwei Shang, Nan Yang, Shujun Pan, Juan Jin, Qiang He

**Affiliations:** ^1^Urology and Nephrology Center, Department of Nephrology, Zhejiang Provincial People’s Hospital, Affiliated People’s Hospital, Hangzhou Medical College, Hangzhou, Zhejiang, China; ^2^Department of Nephrology, the First Affiliated Hospital of Zhejiang Chinese Medical University, Zhejiang Provincial Hospital of Traditional Chinese Medicine, Hangzhou, Zhejiang, China

**Keywords:** kidney, CKD, diagnosis, treatment, nanoparticles

## Abstract

With the development of nanotechnology, nanoparticles have been used in various industries. In medicine, nanoparticles have been used in the diagnosis and treatment of diseases. The kidney is an important organ for waste excretion and maintaining the balance of the internal environment; it filters various metabolic wastes. Kidney dysfunction may result in the accumulation of excess water and various toxins in the body without being discharged, leading to complications and life-threatening conditions. Based on their physical and chemical properties, nanoparticles can enter cells and cross biological barriers to reach the kidneys and therefore, can be used in the diagnosis and treatment of chronic kidney disease (CKD). In the first search, we used the English terms “Renal Insufficiency, Chronic” [Mesh] as the subject word and terms such as “Chronic Renal Insufficiencies,” “Chronic Renal Insufficiency,” “Chronic Kidney Diseases,” “Kidney Disease, Chronic,” “Renal Disease, Chronic” as free words. In the second search, we used “Nanoparticles” [Mesh] as the subject word and “Nanocrystalline Materials,” “Materials, Nanocrystalline,” “Nanocrystals,” and others as free words. The relevant literature was searched and read. Moreover, we analyzed and summarized the application and mechanism of nanoparticles in the diagnosis of CKD, application of nanoparticles in the diagnosis and treatment of renal fibrosis and vascular calcification (VC), and their clinical application in patients undergoing dialysis. Specifically, we found that nanoparticles can detect CKD in the early stages in a variety of ways, such as *via* breath sensors that detect gases and biosensors that detect urine and can be used as a contrast agent to avoid kidney damage. In addition, nanoparticles can be used to treat and reverse renal fibrosis, as well as detect and treat VC in patients with early CKD. Simultaneously, nanoparticles can improve safety and convenience for patients undergoing dialysis. Finally, we summarize the current advantages and limitations of nanoparticles applied to CKD as well as their future prospects.

## 1. Introduction

### 1.1. Nanoparticles

Many diseases originate from pathological changes at the molecular level, such as gene mutations, protein misfolding, and cell dysfunction, caused by microbial infections. These molecules and infectious agents are nanometers in size and may be located in biological systems protected by nanometer-sized barriers (1). Although this definition identifies nanoparticles with dimensions below 0.1 μm or 100 nm, particularly in the area of drug delivery, relatively large (>100 nm) nanoparticles may be required to load a sufficient amount of drug onto the particles (2). With the development of nanotechnology, nanomaterials have been designed as micro particles with different sizes, shapes, and chemical properties to help diagnose diseases and deliver drugs through biological barriers (3, 4). With a decrease in size, the surface area of nanocarriers increases with a higher particle surface energy, which could help them traverse different biological barriers (5, 6). These unique characteristics, which are not observed in macromolecules, enable the use of nanocarriers for novel applications.

Nanoparticles are mainly divided into inorganic-based nanoparticles, which can be designed in various sizes, structures, and geometries, and are ideal candidates for drug delivery and molecular imaging applications (7, 8). Based on their mechanical, electrical, thermal, and physicochemical properties and biological abilities, carbon-based nanoparticles have been widely explored for various applications, including bioimaging, biosensing, and drug delivery (9, 10). Lipid-based nanoparticles are outstanding drug carriers (11–13). Polymeric nanoparticles are divided into nanocapsules and nanospheres and are ideal drug delivery platforms with the ability to optimize therapeutic strategies for vascular aging-related disorders (14, 15). Finally, biomimetic nanoparticles are formed by integrating different biomaterials onto the surfaces of nanoparticles, which enables them to mimic the biological characteristics and roles of native cells (16). Nanoparticles with diameters ranging from 1 to 160 nm can target various parts of the kidney through various mechanisms. Among them, approximately 1-nm nanoparticles can be used to accumulate in the glycocalyx of the glomerular filtration barrier, whereas 80–90 nm nanoparticles can be used to target the mesangium. In addition, larger nanoparticles (>100 nm) can enter the kidney by secreting in peritubular capillaries into the proximal tubules (17–20). Drug delivery through nanoparticles can improve drug bioavailability and cycle half-life and achieve targeted drug delivery by binding specific ligands to the surface of nanoparticles (21). Generally, the treatment of kidney disease requires high drug concentrations, which may lead to adverse reactions and non-specific uptake in other parts of the body (20). For example, tolvaptan is approved for autosomal dominant polycystic kidney disease, which is reported to cause a variety of adverse reactions, including extreme thirst, polyuria, and drug-induced liver damage (22–24). Therefore, the development of kidney-targeting nanoparticles as a therapeutic strategy for kidney diseases has attracted great interest. After reviewing published literature on nanoparticles and patients with CKD, we discuss the application and development prospects of nanoparticles in CKD.

## 2. Auxiliary examination of CKD patients with nanoparticles

### 2.1. Detection of urine toxin gas

Irreversible loss of renal function ultimately makes patients rely on renal replacement therapy such as dialysis or kidney transplantation (25, 26). With the loss of kidney function, toxins cannot be discharged and eventually accumulate in the body (27, 28). According to some reports, hundreds of urine toxins accumulate in the human body (29). At present, renal function in patients with CKD is mainly evaluated based on blood creatinine and urea levels, but these are affected by age, sex, weight, diet, and other factors (30). The diagnosis and treatment of CKD are often delayed because the disease has no obvious symptoms (31). The plasma biomarkers of patients with CKD or some urotoxins in their metabolites are transferred to alveolar exhalation through lung exchange, and even in the late stages of the disease, the patient’s breath has a fishy smell (32). Haick et al. used a carbon nanotube-based sensor to distinguish between healthy and end-stage renal disease rats using breathing samples. This sensor, aimed at the early detection of patients with CKD, can distinguish 27 gases that appear in end-stage renal disease but not in healthy mice (33). In 2012, Marom et al. used a sensor based on organic functionalized gold nanoparticles combined with a support vector machine to analyze respiratory samples. The combination of the three gold nanoparticle sensors could effectively distinguish between early CKD and healthy states (accuracy: 79%) and the fourth and fifth CKD states (accuracy: 85%). This shows that the gold nanoparticle sensor can be used as a fast and reliable diagnostic instrument for the early detection and monitoring of CKD progression (31). Later, a device for measuring human breath ammonia was developed based on polyaniline nanoparticles and used to measure human respiratory ammonia, which could facilitate and lower the cost of monitoring patients with CKD (34). However, these sensors have not been widely used in clinical practice, which may be because their convenience and accuracy require improvement, and recent research on the application of respiratory sensors in CKD is relatively limited (Table 1).

**Table 1:** Examples of clinical studies about different nanoparticles in the CKD.

Nanoparticle type	Purpose	Function	Application and advantages	People	Article
Polyaniline nanoparticle	Inspection	Measuring human breath ammonia was developed based on a single use, disposable, inkjet printed ammonia sensor fabricated using polyaniline nanoparticles	Using low cost point of care breath ammonia systems as a noninvasive means of monitoring kidney dysfunction and treatment.	HD	(32)
Silver nanoparticles	Inspection	Assessing albuminuria in the normal-to-mildly increased albuminuria range	Early detection of slightly elevated urinary microalbumin to prevent the evolution to a large amount of urinary albumin	CKD	(35)
Gold nanoparticles	Inspection	PANI/AuNCs Nanocomposites synergistically improve the immunosensor to detect human serum albumin (HSA) in urine	Chronic kidney disease (CKD) can be detected early by detecting a small amount of human serum albumin (HSA) in urine or microalbuminuria (30–300 μ g/mL).	CKD	(36)
PS/Ag/ab-HSA nanoprobes	Inspection	Detection of microalbuminuria with PS/Ag/ab-HSA nanoprobes	Detection of microalbuminuria is simple, fast, portable and easy to detect CKD early	CKD	(37)
Albumin nanoparticles	treatment	EDTA chelation therapy targeting nanoparticles to reverse CKD related MAC	targeted NP therapy will provide an attractive option to reverse calcification and has a high potential for clinical translation.	CKD	(38)
Silica nanoparticles (SiNP)	Inspection	Silicon oxide nanoparticles (SiNP) with fluorescent anti CD11b as imaging tools	It is deposited in the kidney with inflammation and fibrosis, helping to diagnose the nephropathy	CKD	(39)
Gold nanoparticles	Inspection	Gold nanoparticles conjugated to an anti-collagen-I antibody	It was able to visualize kidney fibrosis *in vitro* and *in situ* and may be useful for nondestructive quantification of tissue fibrosis.	CKD	(40)
Chitosan nanoparticles	Treatment	Nanoparticles expressing bone morphogenetic protein 7 (BMP7) or hepatocyte growth factor (HGF) – NK1 (HGF)/NK1	The transmission of BMP7 reverses the progress of fibrosis and regenerative tubules, and the delivery of HGF/NK1 prevents CKD progression by eliminating collagen fiber deposition.	CKD	(41)
Polyaniline nanoparticle	Treatment	Emodin-NP improves the problems of poor solubility of emodin, limited retention time of colon flushing, insufficient colon adhesion, etc.	Emodin-NP alleviates renal dysfunction and tubulointerstitial fibrosis by regulating intestinal microflora imbalance	CKD	(42)
Albumin nanoparticles	Treatment	Package farnesyl thiosalicylic acid (FTS) to improve its bioavailability	AN-FTS accumulates preferentially in the fibrotic kidney and can significantly reduce renal fibrosis and inflammation than free drugs.	CKD	(43)
Superparamagnetic iron oxide nanoparticle	Treatment	Treatment of anemia by intravenous administration of ferumoxytol	The effect of ferumoxytol by intravenous injection is more convenient and less harmful than that of oral iron	CKD	(44)
Superparamagnetic iron oxide nanoparticle	Inspection	Ferumoxytol enhanced MR angiography for blood vessel examination before creating arteriovenous fistula (AVF) in hemodialysis	Ferumoxytol enhanced MR angiography showed peripheral arterial diseases that could not be identified by duplex ultrasound, and could better predict the outcome of arteriovenous fistula surgery than duplex ultrasound.	HD	(45)
Polyaniline nanoparticle	Treatment	Targeted drug delivery to damaged podocytes	NPs deliver dexamethasone nanoparticles to damaged podocytes for repair	CKD	(46)
Ag nanoparticles and arginine-treated multiwalled carbon nanotubes (MWNT-Arg)	Treatment	MWNT Arg and Ag nanoparticles have good blood compatibility when applied to dialysis membranes	Reduce the adverse reaction of hemodialysis	HD	(47)
gold nanoparticles	Treatment	Gold nanoparticles modified artificial kidney(AuNPs@AK)	It expresses negatively charged surface, which reduces some acute adverse reactions caused by dialysis induced protein adsorption, platelet adhesion and coagulation activation, thus avoiding thrombosis during HD and reducing oxidative stress caused by dialysis.	HD	(48)
Gold nanoparticles	Inspection	Biosensor of Pt Electrode Modified with Gold Nanoparticles (AuNP)	The designed biosensor is used to measure the relative loss of amino acids in patients receiving renal replacement therapy by analyzing the amino acid level in diluted serum samples before and after entering/leaving the hemodialysis device	HD	(49)
Magnetic nanoparticles (MNPs)	Treatment	Magnetic nanoparticles (MNP) as drug carriers to prepare MNPs loaded with vitamin D	MNPs have the same therapeutic effect as vitamin D3 in improving peritoneal fibrosis and functional deterioration, and these particles reduce the side effects of vitamin D3 in mice	PD	(50)
Cationic cellulose nanocrystals (cCNC)	Treatment	cCNC combines with phosphate to reduce blood phosphorus	cCNC phosphate binding rate is higher than that of calcium carbonate and other phosphorus adhesives currently used	CKD	(51)

**Figure 1:**
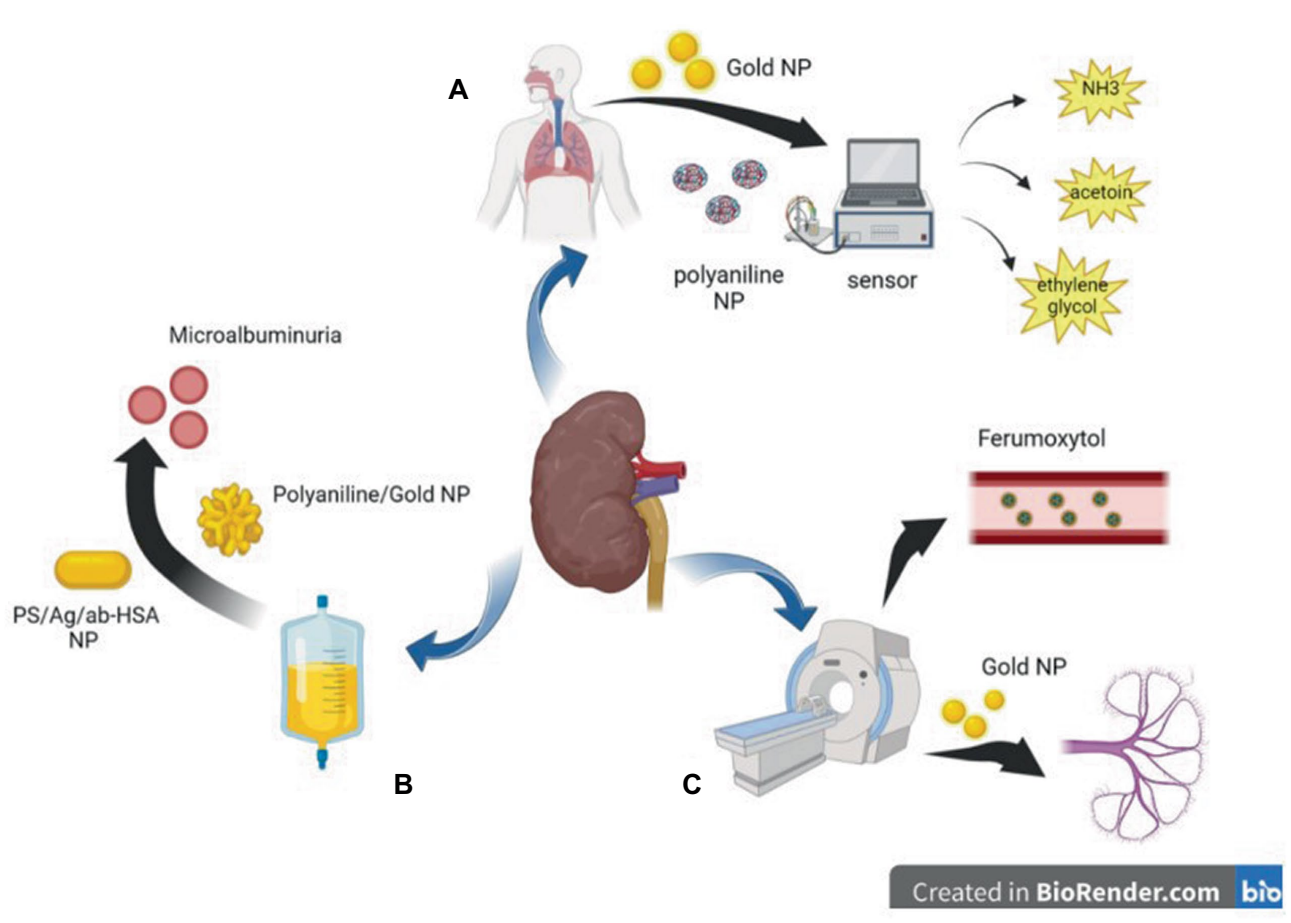
Diagram of application of nanoparticles in auxiliary inspection. **(A)** polyaniline nanoparticles and Gold nanoparticles based sensors can detect harmful gases in the breath of CKD patients. **(B)** Polyaniline nanoparticles and Gold nanoparticles based biosensor can detect trace albumin in the urine of patients with CKD at an early stage. **(C)** Gold nanoparticles and magnetic nanoparticles can be used in CT or MRI imaging to reduce kidney damage caused by iodine or gadolinium contrast agents.

### 2.2. Detection of urinary albumin

Urinary albumin level is a risk factor for CKD or CKD progression (52, 53). However, in recent years, many studies have suggested that urinary albumin excretion below the defined microalbuminuria range (i.e., in the normal-to-mildly increased albumin range) is an independent risk factor for renal disease (54, 55), and some studies have proposed the lowering of albuminuria levels that require intervention from 30 mg/24 h to 8–10 mg/24 h (56, 57). These studies have led to an increasing demand for rapid and easy-to-use screening tests to detect low albumin levels in the general population (35). Biosensors based on nanoparticles have the advantages of simple operation, high sensitivity, good stability, good specificity, fast response, and low analysis cost, among other advantages (58). Surface-enhanced Raman spectroscopy (SERS) is an emission technique using silver/copper/gold surfaces that involves the inelastic scattering of incident laser energy (59)and is highly sensitive, simple, and fast (60). Stefancu et al. developed a SERS-based screening method using iodide-modified silver nanoparticles for assessing albuminuria in the normal-to-mildly increased albuminuria range and showed an excellent correlation between predicted and reference albumin concentrations with an R2 and RMSEP of 0.98 and 2.82, respectively, which highlighted the potential of this strategy for absolute quantification of albuminuria (35). In a recent report on the development of an inexpensive and disposable immunosensor for sensitivity, the carbon working electrode was sequentially modified with electropolymerized polyaniline (PANI) and electrodeposited gold nanocrystals (AuNCs), and the resulting PANI/AuNC nanocomposite synergistically improved the immunosensor response. The PANI/AuNC nanocomposite can detect changes in HSA concentrations in the range of 3–300 μg/mL to screen for early CKD (36, 61). Shaikh et al. developed novel PS/Ag/ab-HSA nanoprobes (polystyrene nanoparticle core with silver nanoshells covalently conjugated to HSA antibodies) and a portable immunosensor for the early detection of CKD (37) (Figure 1).

In general, the use of nanotechnology for urine detection has enabled the development of a new method to detect CKD at an early stage, leading to more efficient treatment monitoring aimed at slowing or even stopping the evolution of macroalbuminuria. Currently, new nanosensors to detect the level of early urinary albumin more sensitively and conveniently, helping to diagnose CKD in the early clinical stages, are still being developed.

### 2.3. CT/MRI imaging contrast agent

Magnetic nanoparticles were developed as angiography agents for molecular imaging in the 1990s (62). The magnetic particle imaging signal is only generated by magnetic nanoparticles, and diamagnetic tissue generates a zero background signal to produce better vascular contrast (63, 64). Iodine and gadolinium contrast agents for X-ray imaging and magnetic resonance imaging can further lead to renal fibrosis, and nephrogenic systemic fibrosis is recognized as a serious complication related to gadolinium contrast agents; therefore, the use of these examinations in patients is restricted (65–67). Unlike other contrast agents, iron is an essential element for the human body; therefore, a good choice for patients with CKD is to use ultra-small superparamagnetic iron oxide nanoparticles, such as ferumoxytol, as contrast agents (68). Although feumoxytol nanoparticles have an ultra-small core, their large carbohydrate-coated shell prevents renal filtration, and they are removed from the circulatory system through phagocytosis by macrophages. The remaining iron oxide particles are absorbed by the reticuloendothelial system of the liver, spleen, and bone marrow and are incorporated into iron storage in the body for red blood cell synthesis, thus prolonging the stay time in blood vessels and allowing for a more lasting contrast (64, 69–72). Therefore, it is relatively safe for patients with renal failure (73) and can be considered for patients with a glomerular filtration rate ≤ 30 mL/min/1.7 (74). Some studies have shown that ferumoxytol can also be used as a contrast agent for clear imaging in children who have undergone dialysis, and the imaging results are consistent with the real anatomy, with good diagnostic effects and no adverse events; thus, it is expected to replace other contrast agents and be widely used in patients with CKD (75, 76).

Yu et al. recently used renal-clearable near-infrared-emitting glutathione-coated gold nanoparticles (GS-AuNPs) as contrast agents in fluorescence imaging to evaluate and determine the stage of renal dysfunction, which is consistent with renal injury evaluated by pathological results (77). Previously, they applied GS-AuNPs to the fluorescence imaging of renal clearance kinetics in normal mice to prove that they are non-toxic and do not affect metabolism *in vivo* (78). The hydrodynamic diameter of GS-AuNPs (3.3 ± 0.4 nm) is below the renal filtration threshold (6–8 nm), so they can be effectively excreted through the kidneys (79). At present, many nanoparticles can be filtered by the kidney, including gold nanoparticles, copper nanoparticles (80), iron oxide nanoparticles (81), silicon dioxide nanoparticles (82), carbon dots (83), and palladium nanoparticles (84), making the application of nanoparticles in non-invasive renal imaging possible.

## 3. Nanoparticles and vascular calcification

Vascular calcification (VC) is a risk factor for cardiovascular events and death in patients with CKD (85, 86). VC formation is caused by multiple factors, such as the proliferation, differentiation, and apoptosis of vascular smooth muscle cells (VSMC), oxidative stress, endothelial dysfunction, increased remodeling of the extracellular matrix, release of calcified extracellular vesicles, loss of mineralization inhibitors, and chronic inflammation (87). High P content can induce the formation of Ca/P-related nanocrystals in the cell matrix. The presence of these crystals leads to an increased expression of osteoblast bone morphogenetic protein 2 (BMP-2) and osteopontin. Among these, Ca/P crystals with diameters of 1–2 nm or less are swallowed by VSMCs, causing toxic effects. The dissolution of crystals in the lysosomes leads to the release of calcium ions into the cytoplasm, resulting in apoptosis (88). Nanocrystalline Ca/P can affect a variety of cell functions, including proliferation, activation of the mitogen-activated white kinase signaling cascade, activation of matrix metalloproteinases, induction of proinflammatory cytokines, and stimulation of endocytosis (89–91). Therefore, the presence of nanocrystalline Ca/P may lead to a pathological positive feedback loop, resulting in increased cell death, inflammation, phenotypic changes, matrix degradation, and calcification (92, 93). Schlieper et al. collected iliac artery segments from 30 patients undergoing dialysis before kidney transplantation and showed that many calcifications consisted of 2–10 nm nanocrystals with a hydroxyapatite and whitlockite crystalline structure and a mineral phase (94). This confirms that the VC may have originated from nanocrystals formed by mineral deposition.

**Figure 2:**
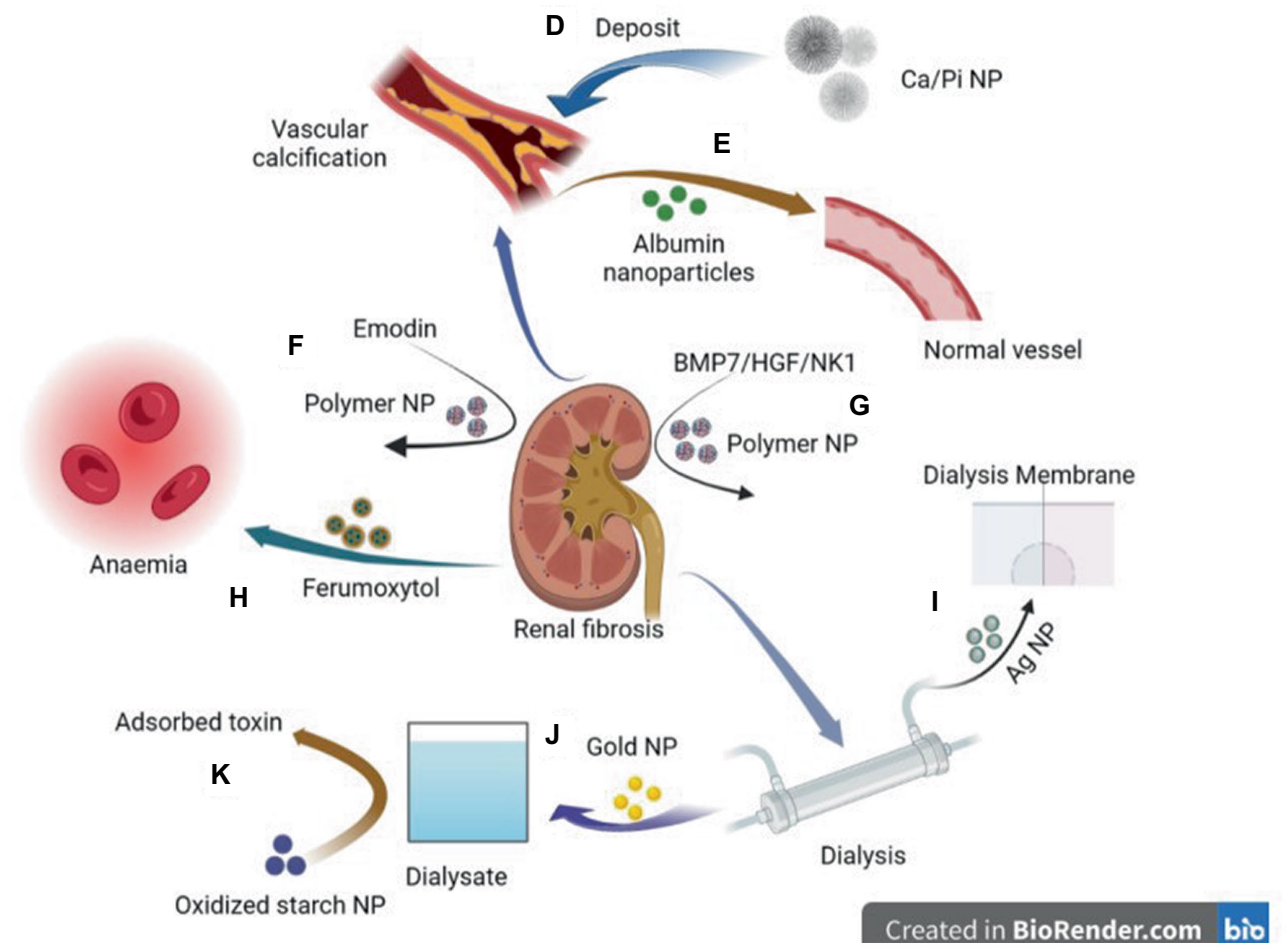
Diagram of application of nanoparticles in various comorbidities caused by chronic kidney disease. D: Deposition of CA/ Pi-related nanocrystalline compounds in the vascular wall leads to vascular calcification. E: Elastin antibodies to albumin nanoparticles can reverse CKD—associated MAC. F: Emodin-NP regulates gut microbiome and delays the progression of CKD. G: Plasmid DNA of BMP7 or HGF/NK1 encapsulated in chitosan nanoparticles coated with hyaluronic acid reversed fibrosis progression of the cell and regenerative tubules. H: Ferumoxytol is a novel, semi-synthetic, carb-coated, superparamagnetic iron oxide nanoparticle that can be administered intravenously for iron supplementation. I: Ag nanoparticles modified dialysis membranes to reduce thrombosis. J: A biosensor based on a gold nanoparticle (AuNP) modified Pt electrode can measure relative amino acid loss in patients undergoing renal replacement therapy. K: Oxidized starch nanoparticles can adsorb and remove 95% urea in dialysate.

Calcium phosphate overloading in the human body induces CKD (95, 96). Increased phosphate excretion per nephron increases phosphate concentration in renal tubular fluid and triggers CaPi crystal precipitation (97). The CaPi precipitate may combine with fetuin-A to form calcium protein granules (CPP). CPP deposits in the proximal renal tubule epithelium and induces the destruction of lysosome homeostasis, autophagic flux, and plasma membrane integrity, resulting in a vicious cycle and finally leading to progressive nephron loss (98). Pasca et al. described a nanoparticle-based assay that, in the presence of artificially elevated calcium and phosphate concentrations, detects the spontaneous transformation of spherical colloidal primary calciprotein particles to elongate crystalline secondary CPPs. Using this assay, we found that both the sera of mice deficient in fetuin-A, a serum protein that inhibits calcification, and the sera of patients on hemodialysis had reduced intrinsic properties to inhibit calcification (99). Karamched et al. developed elastin antibody-conjugated albumin nanoparticles that target only degraded elastin in the vasculature while sparing healthy tissues. Targeted nanoparticle-based ethylenediaminetetraacetic acid chelation therapy to reverse CKD-associated Medial arterial calcification (MAC) and targeted nanoparticle therapy may provide an attractive option to reverse calcification and have a high potential for clinical translation (38) (Figure 2).

The VC material of CKD has been confirmed to exist in the form of nanoparticles, and experimental research has shown that albumin nanoparticles with elastin antibodies have the potential to reverse vascular calcification, which is important for clinical applications. However, at present, there are few results and insufficient evidence. Thus, the application of nanotechnology in the diagnosis and treatment of VC in patients with CKD requires more basic research to confirm its utility. Thus, this is a promising direction for future research.

## 4. Nanoparticles and renal fibrosis

Fibrosis is an adverse repair reaction of extracellular matrix deposition after tissue damage that may eventually lead to organ dysfunction (100, 101). Progressive fibrosis is a potential pathophysiological process of CKD (102), which is closely related to inflammation (103, 104), secondary hyperparathyroidism (105), FGF23 (106, 107), diabetes (108), vitamin D deficiency (109), and other factors. Determining the degree of renal fibrosis is conducive to monitoring disease progression and management. Currently, renal biopsy is the reference standard for evaluating fibrosis; however, its application is limited owing to its invasivity (110). There is a great need to develop non-invasive imaging methods for the evaluation of renal fibrosis, and magnetic resonance (MR) with or without gadolinium enhancement shows great potential (111). However, gadolinium or iodine administration for MR and computed tomography (CT) angiography is not applicable in all cases and introduces the risk of contrast agent nephropathy (112). Therefore, researchers have begun to explore the application of nanoparticles in the diagnosis of renal fibrosis. Shirai et al. developed silica nanoparticles with fluorescent-labeled anti-CD11b as an imaging tool to efficiently evaluate inflammation and fibrosis in an animal model of unilateral ureteral obstruction (39). In addition, based on the fact that collagen-I is one of the main components of pathological fibrosis, selectively directing gold nanoparticles (AuNP) to collagen-I may help identify damaged kidneys (113). Zhu et al. (40) combined an anti-collagen-I antibody to the surface of AuNPs and injected them into mouse arteries. After 4 h of CT evaluation, the retention of Co-I-AuNPs in the kidney on the side of fibrosis increased. These experiments showed that nanotechnology can be used to detect renal function and study pathologies in the future.

In addition, it is important to develop treatment methods for CKD to prevent or reverse the fibrogenic cell phenotypes. Nanoparticles can provide new methods of anti-fibrosis therapy for damaged tissues and resident cells by limiting the expression of pro-fibrotic phenotypes.

The size and surface properties of polymer nanoparticles can be adjusted to help deliver drugs to specific sites; for example, loading dexamethasone nanoparticles into selected biocompatible nanoparticles successfully repaired damaged podocytes (114). They can be used to modify drugs to achieve improved stability, durability, and therapeutic effects. For example, emodin, a traditional Chinese medicine, can regulate intestinal microbiota and delay CKD progress (115, 116). However, poor solubility, limited colonic irrigation retention time, and inadequate colon adhesion hinder the clinical application of emodin. Lu et al. (42) prepared monomethoxy-poly (ethylene glycol)-poly (lactic acid)-chitosan-2-mercaptobenzimidazole nanoparticles that incorporated emodin (emodin-NPs). Emodin-NP has higher stability and better colonic adhesion and durability than emodin alone, and can alleviate kidney dysfunction and tubulointerstitial fibrosis by modifying gut microbiota disorders. In addition, nanoparticles can be used to decorate small molecules and deliver them to specific cells to play an anti-fibrotic role. For example, plasmid DNA expressing bone morphogenetic protein 7 or hepatocyte growth factor (HGF)-NK1 (HGF/NK1) was encapsulated in nanoparticles to safely administer multifunctional nanoparticles containing plasmid DNA to the kidneys for localized and sustained expression of antifibrotic factors (41). In addition, nanoparticles can improve the water solubility and bioavailability of molecular substances. Farnesyl thiosalicylic acid (FTS) is a specific inhibitor of the proto-oncogene Ras. It has been reported that FTS can inhibit Transforming growth factor (TGF) by inhibiting the EMT process-β 1 induced activation of NRK-49F in renal fibroblasts and may have therapeutic potential in treating renal fibrosis (117–120). However, its application is hindered by its water insolubility and low bioavailability. Huang et al. encapsulated FTS in bovine serum albumin nanoparticles (AN-FTS) to improve its characteristics. Mouse experiments have demonstrated that AN-FTS preferentially accumulates in the fibrotic kidney, which can significantly reduce renal fibrosis and inflammation compared to free drugs (43).

In general, nanotechnology can not only evaluate renal fibrosis in combination with CT, but also treat renal fibrosis owing to its advantages of strong plasticity, targeting, and stability. It can improve the bioavailability of drugs and reduce their adverse effects. Therefore, the application of nanoparticles has opened a new chapter in the treatment of CKD and has enormous potential for the treatment of renal fibrosis.

## 5. Nanoparticles and renal anemia

Oral or intravenous iron drops are primarily used to treat renal anemia. Ferumoxytol is a new, semisynthetic, carbohydrate-coated, superparamagnetic iron oxide nanoparticle that can be administered intravenously. It produces the least dialyzable free iron and can release free iron more stably than the other iron products. Therefore, its bioavailability is better than that of oral administration, and its tolerance is better, with fewer side effects (44, 70, 121). Many randomized controlled trials and cohort studies have shown that it is more effective than oral iron in improving anemia and can increase transferrin saturation (TSAT) and ferritin levels. The probability of most adverse events is similar to that of other oral iron (122–128). A higher risk of hypotension may be associated with fewer adverse gastrointestinal events (129). However, Ferumoxytol received a black box warning from the FDA in March 2015, reminding doctors to pay attention to the possibility of hypersensitivity reactions, including allergic reactions. Therefore, it is necessary to reduce the infusion rate.

## 6. Application of nanoparticles in dialysis

Renal failure was defined as a glomerular filtration rate of <15 mL/min/1.73 m^2^ (130). Dialysis is one of the main treatment methods for patients with renal failure (131), and the number of dialysis patients has gradually increased over the last 20 years (132). Although dialysis treatment has become increasingly developed, the mortality rate of patients undergoing dialysis remains very high (133). Various complications may arise as a result of dialysis, such as cardiovascular events, catheter-related infections, peritonitis, among others (134–136). In addition, dialysis may cause great inconvenience to patients, especially those undergoing hemodialysis, because a dialysis frequency of three times/week may result in patients risking unemployment to attend dialysis appointments (137). The increase in economic burden causes the disease burden on patients to rise continuously (138); thus, our goal is to make dialysis technology safer, more efficient, and more convenient.

Nanotechnology can be used as a new method for treating arteriovenous fistulas. Ferumoxytol is an iron-oxide nanoparticle that can be used as a substitute for MRI contrast agents. Ferumoxytol can identify the condition of the central artery, better identify peripheral artery diseases, and better predict the results of arteriovenous fistula surgery (45).

One of the main obstacles to blood filtration is blockage caused by thrombosis, which limits the maximum filter life during hemodialysis and continuous renal replacement therapy (46). Dialysis membrane pores must be sufficiently small to prevent protein loss from plasma, and membrane surfaces should be carefully designed to provide high membrane blood compatibility and minimal thrombosis (47). Engineering changes in membrane surface chemistry and structure by doping with nanomaterials has attracted extensive attention. Ag nanoparticles and multiwalled carbon nanotubes (MWNT) may be the most important nanomaterials with blood compatibility and low toxicity to blood cells (47). For example, a gold nanoparticle-modified artificial kidney, which expresses a negatively charged surface, reduced some acute adverse effects caused by dialysis-induced protein adsorption, platelet adhesion, and coagulation activation, thus avoiding thrombosis during hemodialysis (48). In addition, an AuNP-modified Pt electrode was used to measure the relative loss of amino acids in patients undergoing renal replacement therapy by analyzing the amino acid levels in diluted serum samples before and after entering/leaving the hemodialysis apparatus (49).

Nanomaterials have the advantage of enhanced accumulation in the target area and can be used in peritoneal dialysis treatment. Zheng et al. (50) developed vitamin D-loaded magnetic nanoparticles (MNPs), Vitamin D-loaded MNPs targeted the peritoneum better than vitamin D3 and had the same therapeutic effect as vitamin D3 in ameliorating peritoneal fibrosis and functional deterioration in a peritoneal dialysis animal model. In addition, nanomaterials as adsorbents are expected to facilitate portable dialysis. A nano-adsorbent derived from corn starch (oxy-SNPs) for urea removal and adsorption achieved equilibrium after 4 h, with 95% removal (139). Thus, oxy-SNPs are promising adsorbents for dialysate regeneration and urea removal. In addition, cationic cellulose nanocrystals are also used as phosphate binders to treat patients with hyperphosphatemia, and their phosphate binding rate is higher than that of commonly used phosphate binders, such as calcium carbonate (51).

In general, nanomaterials can be used in various fields for patients undergoing dialysis, such as optimizing dialysis effects, reducing adverse reactions to dialysis, evaluating dialysis conditions, and treating dialysis-related complications. However, most of these studies have been limited to mouse models.

## 7. Limitations of nanoparticle application

However, despite our increased exposure to nanoparticles, information regarding nanoparticle safety lags behind research on the application of nanoparticles (140). The toxic effects of nanoparticles on tissues and organs should not be ignored. In nanomedicine, a large amount of drugs needs to accumulate in target cells to achieve a therapeutic effect (141), and through the blood circulation, nanoparticles can be distributed and can accumulate in different organs such as the liver, spleen, lungs, and kidneys. Some studies suggest that nanoparticles may also accumulate in the brain if they are small enough (<10 nm) and/or the blood–brain barrier is not intact (142, 143). The toxicity of nanoparticles is dependent on their biophysical properties, including size, surface area, surface charge, and aggregation state (144). These properties have been shown to affect their distribution and deposition in different organ systems (145). Solid nanoparticles such as metal-containing or metal oxide nanoparticles have reportedly caused oxidative stress, inflammation, and DNA damage, and they have been shown to induce oxidative stress in the liver, spleen, and kidneys and inhibit the effects of antioxidants (146–148). At present, most studies evaluating the toxicity of nanoparticles are short-term exposure studies, and few studies have evaluated the toxic effects of nanoparticles after chronic exposure (149). In addition, the pharmacokinetics of nanoparticles as drug delivery systems are not well studied, and most studies have focused only on the pharmacokinetics of encapsulated drugs as opposed to the drug carriers themselves (150, 151).

Although attempts have been made to modify nanoparticle surface properties using inaccessible coating materials to reduce their potential toxicity, this may alter the cellular uptake of nanoparticles and their efficacy as a drug delivery system (152, 153). Therefore, the toxic effects of nanoparticles limit their clinical application (154), and approximately 20% of the nanoparticles rejected during clinical trials are for safety reasons (149). In the future, a complete toxicological analysis of nanoparticles and the development of structure–activity relationships will help determine the key physical or chemical characteristics of nanoparticles that cause their toxicity. By optimizing their physical and chemical characteristics, the toxicity of nanoparticles can be minimized and their biological effects can be maximized (155, 156).

## 8. Conclusion

The emergence of nanoparticles has brought hope to medicine. Regarding kidney disease, early potential patients and disease progression can be identified using gas measurements and urine detection. Magnetic nanoparticles can reduce the renal fibrosis caused by iodine or gadolinium contrast agents in patients with CKD. Gold nanoparticles and silica nanoparticles are also used for renal imaging to evaluate renal inflammation and fibrosis, and may become a tool to replace renal biopsy puncture in the future. CKD is associated with vascular and renal fibrosis. Albumin nanoparticles can target degraded elastin to reverse vascular calcification. While polymer nanoparticles can package and deliver related drugs to specific parts of the kidney to improve their bioavailability and half-life and effectively treat renal fibrosis, superparamagnetic iron oxide nanoparticles can be administered intravenously to treat renal anemia and appear to be more effective than traditional oral iron agents. In addition, during dialysis treatment, the use of gold nanoparticles can improve the chemical structure of the dialysis membrane, thus reducing the adverse reactions of thrombosis and detecting the loss of amino acids in patients after dialysis. In summary, many types of nanoparticles have been used for the diagnosis and treatment of patients with CKD; however, most trials are still in the preclinical stage, and nanoparticles have not been routinely used in clinical practice. We believe that the application of nanoparticles to patients with CKD is clinically meaningful, as it may allow for more accurate and effective diagnosis and treatment of CKD in the future. Therefore, more clinical experiments on nanoparticles should be conducted so that nanotechnology can be more widely and effectively utilized in clinical settings.

## Author contributions

KY drafted and revised the manuscript. YS drafted and revised the manuscript and designed the tables/figures. SP and NY helped collect data. JJ drafted and revised the manuscript. QH initiated the collaboration, directed, drafted, and revised the manuscript.

## Funding

This research was supported by the Huadong Medicine Joint Funds of the Zhejiang Provincial Natural Science Foundation of China (grant no. LHDMZ22H050001), Construction of Key Projects by the Zhejiang Provincial Ministry (project no.WKJ-ZJ-2302), Zhejiang Province Chinese Medicine Modernization Program (project no. 2020ZX001), Key Project of Scientific Research Foundation of Chinese Medicine (2022ZZ002), Key Project of Zhejiang Science and Technology Department (2022C03118), and Key Project of Basic Scientific Research Operating Funds of Hangzhou Medical College (KYZD202002).

## Conflict of interest

The authors declare that the research was conducted in the absence of any commercial or financial relationships that could be construed as a potential conflict of interest.

## Publisher’s note

All claims expressed in this article are solely those of the authors and do not necessarily represent those of their affiliated organizations, or those of the publisher, the editors and the reviewers. Any product that may be evaluated in this article, or claim that may be made by its manufacturer, is not guaranteed or endorsed by the publisher.
